# Cholera—How to Mitigate It

**Published:** 1865-11

**Authors:** 


### Cholera—How to Mitigate it.

At the first meeting of the *American Association for the Promotion of Social Science*, held in Boston on the 4th of October, Dr. H. G. Clark read a paper from which the following extract is taken:

"Of all other plagues, cholera has taken the widest geographical and climatic ranges. It has visited at all seasons all quarters of the globe, and has in the most extraordinary manner overleaped all ordinary bounds. It has spread without regard to quarantine laws or cordons, touching emigrant ships in mid-ocean a thousand miles apart and landing with them at their ports of destination. Its first visitation to this country was in 1837, when it prevailed to a moderate extent. In the month of September, 1832, the disease disappeared, but revisited us with renewed violence in the winter of 1848 and the summer of 1849. It first manifested itself at Staten Island, on the 2d of December, and nine days later at New Orleans. From these two commercial centres it spread over the whole country. The epidemic was not general till about the first day of May, and and had terminated by the last of November, having destroyed in the large towns (which only were reported) thirty thousand persons. An epidemic of very moderate intensity occured in 1853—about one-tenth that of 1849.

"The epidemic now prevailing in Europe has spread much more rapidly than has been its wont, while the mortality has been fearful. In Lower Egypt, 80,000 have died. So far only, a few sporadic cases have occurred in this country, but its arrival, judging from the past, is only a question of time. To the measures necessary to be adopted, if we wish to escape from, or to diminish the intensity of the expected attack, there are two parties. First, the various boards of health, their constitutional advisers and their executive officers. Second, the masses of the people, especially those of the laboring classes. The co-operation of both these classes is indispensable. By intelligent combined action all epidemic diseases, especially cholera, can be very much, controlled, and their ravages stayed. On the important point of how this is to be accomplished, Dr. Clark made the following important sug gestions:—

"Shall we, as some distinguished civilians and others have proposed, establish a rigid quarantine? Shall we exclude all vessels and persons and goods from the Mediterranean from our ports? Shall we obstruct the course of trade in its accustomed channels by land and sea? No—a thousand times, no! If this were possible—if all these and more were possible—how could it avail to arrest the march of a pestilence that walketh in darkness, and that springs out of the earth by intangible but noxious exhalations which are wafted to us without any known agencies, on the wings of the wind, over continents and oceans?

"But above all, when we consider the now established and well-proven fact, that colera is neither contagious nor infectious, how doubly absurd do quarantines appear? Let us therefore dismiss all our fears on this point, relieve ourselves and the authorities from the annoyances and evils incident to a rigorous quarantine, and apply ourselves to the only available but powerful and sufficient remedy to be found in the proper use and application of intelligently directed sanitary measures, and to energetic administration, by the boards of health, under the advice of their medical officers, of all the provisions of sanitary law. These duties comprise, in a great city, all the departments usually comprised under the titles of external and internal health, and imply attention to the following points, viz:—1, sanitory survey; 2, general cleaning operations; 3, sewerage; 4, *abattoirs* (slaughter houses) and markets; 5, dram shops and drinking-houses; 6, lodging-houses, cellar habitations, tenement-houses, streets and construction of houses, water-supply, ventilation, vaccination, public baths, and interment of the dead, and special care in cases of the presence of epidemic and contagious diseases.

"The subject of dram shops and drinking-houses it may be thought is improperly introduced, but when we all know that, in addition to the pernicious effects of intemperance, the first and most numerous victims to epidemics are the intemperate, it will appear otherwise. Indeed, if, instead of treating and hopelessly attempting to cure and arrest intemperance and dram-selling as crimes and civil offences, and amenable only to civil, criminal, or moral laws, we would treat the first as a disease and the second as a nuisance, and dangerous to the public health, and to be abated as such, we should I am sure, have entered upon the right track; for, instead of the prejudices of the people being excited against a sumptuary law, we should, in favor of a sanitary measure, have their entire sympathy and support.

"The duties of the people themselves may be summed up in short to be:—1. To lay aside all unnecessary fears, and to feel that danger looked full in the face will either be diminished or disappear; 2d. To practice personal and houshold cleanliness; 3. To see that their cellars, drains, and other premises are clean and in order; 4. To avoid over-crowding and to attend to ventilation; 5. To avoid intemperance and the habitiual use of spirituous drinks; 6. To avoid all decayed or unripe fruit"—*Boston Med., and Surg. Journal.*

